# Real-world data on Pressurized IntraPeritoneal Aerosol Chemotherapy (PIPAC)-directed therapy in patients with peritoneal metastases; Third annual report from the ISSPP PIPAC database

**DOI:** 10.1515/pp-2025-0013

**Published:** 2025-06-10

**Authors:** Magnus Skov Jørgensen, Pernille Schjødt Hansen, Claus W. Fristrup, Martin Hübner, Jimmy So, Anne-Cecile Ezanno, Peter Hewett, Miguel Ruiz-Marin, Günther A. Rezniczek, Özgül Düzgün, Marc Pocard, Francesco Casella, Laura Lay, Marisa Aral, Tarkan Jäger, Felix Laminger, Oliver Glehen, Claire-Angéline Goutard, Laurent Villeneuve, Andrea Di Giorgio, Michael Bau Mortensen

**Affiliations:** Odense PIPAC Center (OPC) and Odense Pancreas Center (OPAC), 11286 Odense University Hospital, Odense, Denmark; Department of Surgery, Odense University Hospital, Odense, Denmark; Lausanne University Hospital CHUV, University of Lausanne (UNIL), Lausanne, Switzerland; Surgery, National University Hospital, Singapore, Singapore; Department of Surgery, Hôpital d’Instruction des armées Begin, Saint-Mande, France; Department of Surgery, The Queen Elizabeth Hospital, Woodville South, South Australia, Australia; Department of Surgery, Reina Sofia University General Hospital, Murcia, Spain; Obstetrics & Gynecology, Marien Hospital Herne, Klinikum der Ruhr-Universität Bochum, Herne, Germany; Department of Surgical Oncology, İstanbul Ümraniye Training and Research Hospital, Istanbul, Türkiye; Department of Digestive, Hepatobiliary Surgery and Liver Transplantation, Pitié-Salpêtrière Hospital, Assistance publique-Hôpitaux de Paris, Paris, France; Université Paris Cité, Paris, France; General and Upper GI Surgery, University of Verona, Verona, Veneto, Italy; Department of Gynecology Surgical Area at the Institute of Oncology A. H. Roffo, University of Buenos Aires, Buenos Aires, Argentina; General Surgery Department, Centro Hospitalar Universitário de São João, Porto, Portugal; Department of Surgery, Paracelsus Medical University, Salzburg, Austria; Department of Surgery, Center for Peritoneal Carcinomatosis, Hanusch-Krankenhaus, Vienna, Austria; Service de Chirurgie Digestive et Oncologique, Hôpital Lyon Sud, Hospices Civils de Lyon, Lyon, France; Surgical Unit of Peritoneum and Retroperitoneum, Fondazione Policlinico Universitario A. Gemelli IRCCS, Rome, Italy

**Keywords:** database, ISSPP, PIPAC, peritoneal metastasis

## Abstract

**Objectives:**

In 2020, the International Society for the Study of the Pleura and Peritoneum (ISSPP) launched a database monitoring real-world data on Pressurized IntraPeritoneal Aerosol Chemotherapy (PIPAC)-directed therapy in patients with peritoneal metastases (PM). This study covers data from the third annual report on the ISSPP PIPAC database.

**Methods:**

Systematic analysis of all data reported to the ISSPP PIPAC database between June 15th, 2020, and November 1st, 2024. We hypothesize that ISSPP PIPAC data align with existing literature.

**Results:**

Seventeen PIPAC centers reported 3224 PIPAC treatments in 1126 patients with PM (median number of treatments 2, range 1–33). The median peritoneal cancer index (PCI) at PIPAC 1 was 19 and remained unchanged during subsequent treatments. The number of patients with >500 mL ascites significantly decreased from the first three PIPAC treatments to PIPAC 4+ (p<0.01). Major complications (Dindo–Clavien ≥3b) occurred in 0.7 % of the treatments, while Common. Terminology Criteria for Adverse Events (CTCAE) grades ≥3 were reported in 5.2 %. Peritoneal regression grading score (PRGS) was performed in 2306 (72 %) of the treatments. At PIPAC 1, 2, and 3, complete or major response (mean PRGS ≤2) was achieved in 57 %, 72 %, and 75 % of the patients, respectively. Median overall survival from PIPAC 1 was 12.5 months. Patients with complete/major response (mean PRGS ≤2) at PIPAC 1-3 had a longer overall survival compared to patients with minimal/no response (mean PRGS >2).

**Conclusions:**

This study from the ISSPP PIPAC database provides substantial real-world data demonstrating the feasibility, safety, and potential effect of PIPAC-directed therapy in patients with PM.

## Introduction

Peritoneal metastasis (PM) is a common end-stage disease in patients with gastrointestinal or gynecological cancers. Patients with PM suffer from ascites, bowel obstruction, general fatigue, and reduced quality of life [1], 2]. Systemic chemotherapy has limited effect on PM and most patients have a bad prognosis [3]. Pressurized IntraPeritoneal Aerosol Chemotherapy (PIPAC)-directed treatment was introduced a decade ago as a palliative treatment in patients with PM, striving to overcome some of the limitations of systemic chemotherapy [4].

In 2020, PIPAC reached stage 2b of the IDEAL framework, and a prospective international PIPAC database was launched by the International Society for the Study of the Pleura and Peritoneum (ISSPP). Information about the database and initial results have been published by the first two annual ISSPP PIPAC reports [5], 6]. These annual reports, together with several retrospective as well as phase 1 and phase 2 studies, have provided insights into the potential oncological application and effects of this treatment platform [7]. However, due to lack of comparative studies, PIPAC is still considered an experimental treatment. The ISSPP PIPAC database is, therefore, considered essential for monitoring real-world data in terms of indications, safety, and potential effects of PIPAC, while awaiting data from randomized controlled trials.

This study covers the third annual report from the ISSPP PIPAC database with an updated summary of all recorded data between launch of the database in June 2020 and November 1st, 2024. Registered data were analyzed and monitored in light of present PIPAC-related evidence.

## Methods

This study analyzed data entered into the prospective ISSPP PIPAC database between the official launch on June 15th, 2020, and November 1st, 2024.

A general overview of reporting centers and patient characteristics was combined with a focus on procedure-related complications, response evaluation, follow-up, and survival. The peritoneal regression grading score (PRGS) or an unspecific “non-PRGS” classification was used for response evaluation. Surgical complications were graded using the Dindo–Clavien classification (DC) [8], and adverse events were graded using the Common Terminology Criteria for Adverse Events (CTCAE) version 5.0. We hypothesize, that data from the ISSPP PIPAC database aligns with existing literature in terms of patient selection, procedures, complications, and treatment outcomes.

Information regarding the ISSPP PIPAC database’s software, funding, governance, legal aspects, variables, and implementation have been described previously [6]. In short, the ISSPP PIPAC database is based on a REDCap platform [9] funded and hosted by Open Patient data Explorative Network (OPEN), Odense University Hospital, Odense, Denmark, in collaboration with Odense PIPAC Center (OPC).

### Statistics

Data were analyzed using STATA-software version 17. Descriptive data were presented as numbers and percentage for categorical variables and as medians or means for continuous variables. The χ^2^-test was used for categorical variables, while the Mann–Whitney U test and Fisher’s exact test were applied for continuous variables to calculate differences between groups. Survival data were analyzed using the Kaplan–Meier approach and log rank test. Statistical significance was defined as a p-value ≤0.05 from a two-sided test.

### Ethics

All reporting centers must obtain oral and written patient consent before inclusion of patients into the ISSPP PIPAC database. Furthermore, each center must follow local ethical regulations.

The ISSPP PIPAC database and the study protocol were approved by the Region of Southern Denmark, the Institutional Review Board of the University of Southern Denmark and OPEN in 2020 [6].

## Results

### PIPAC centers

**Figure 1:**
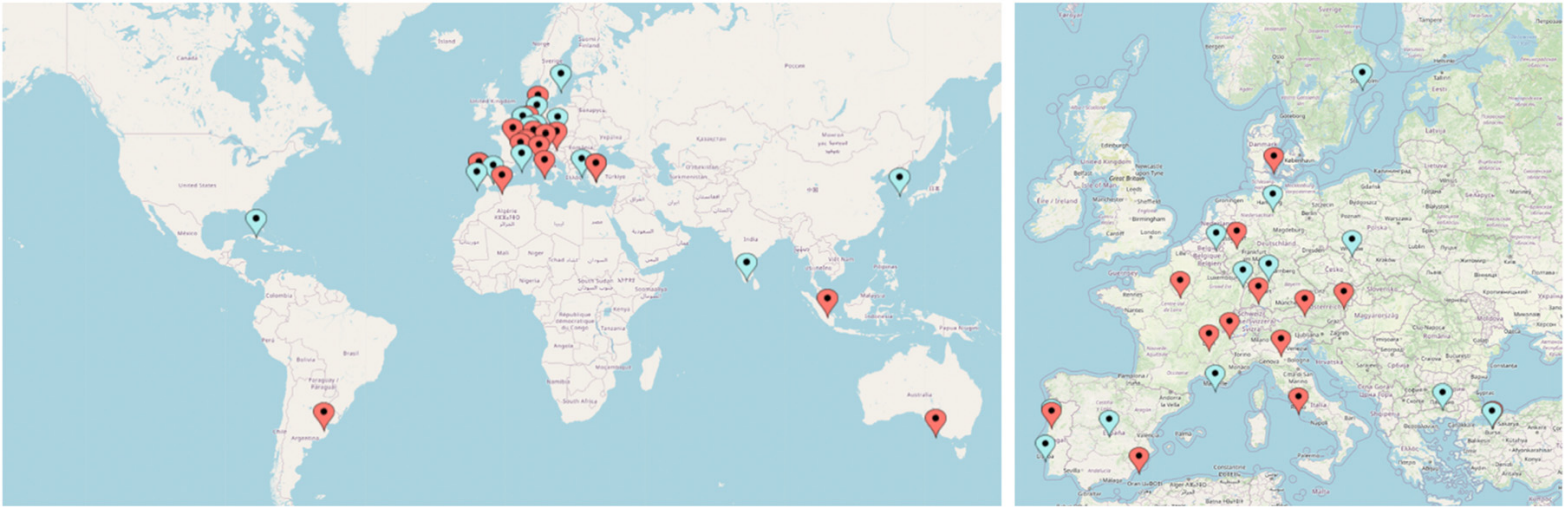
PIPAC centers registered in the ISSPP PIPAC database worldwide (left) and in Europe (right). Red dots represent centers actively reporting patient data. Blue dots indicate centers that have not yet reported patient data.

The ISSPP PIPAC database contained 1126 patients who were treated with 3224 PIPAC-directed treatments as of November 1st, 2024. Thirty-one PIPAC centers were registered, of which 17 (55 %) had reported data to the database during this time (Figure 1).

### Patients

The number of included patients per contributing center ranged between 3 and 390. Four PIPAC centers were responsible for 77 % of the reported patients. Baseline characteristics of all patients are listed in Table 1.

**Table 1:** Demographic data, primary tumor origin, and previous treatments of the 1126 patients included in this study.

Basic characteristics	Total
Total number of patients (%)	1126 (100)
Age, median years (range)	61 (22–89)
Gender, male n (%)	513 (46)
ECOG^a^ performance status, n (%)	
0	309 (27)
1	623 (55)
2	112 (10)
3	10 (1)
Unknown/missing	72 (6)
Primary tumor, n (%)	
Gastric	404 (36)
Colon	181 (16)
Ovaries	141 (13)
Pancreas	91 (8)
Appendix	79 (7)
Peritoneal mesothelioma	62 (6)
Bile duct	47 (4)
Small bowel	19 (2)
Rectum	17 (2)
Breast	16 (1)
Primary peritoneal	15 (1)
Esophagus	9 (0.8)
Uterus	6 (0.5)
Fallopian tube	3 (0.3)
Unknown	11 (0.9)
Other	25 (2)
Histology of primary tumor, n (%)	
Adenocarcinoma	510 (45)
Signet-ring-cell carcinoma	231 (21)
Mucinous adenocarcinoma	102 (9)
Ovarian	67 (6)
Peritoneal mesothelioma	58 (5)
Pseudomyxoma peritonei	27 (2)
Breast	12 (1)
Other	52 (5)
Unknown/missing	67 (6)
Time from diagnosis of primary tumor to PM^b^ (%)	
0–2 months	707 (63)
3–5 months	49 (4)
≥6 months	286 (25)
Unknown/missing	84 (7)
Extraperitoneal metastasis at inclusion, n (%)	94 (13)
Primary tumor in situ, n (%), (n=727)	398 (55)
Previous oncological treatment, n (%)	
Systemic chemotherapy	967 (86)
Immunotherapy	51 (5)
Radiotherapy	20 (2)
Other	62 (6)

^a^ECOG, eastern cooperative oncology group; PM^b^, peritoneal metastases.

### PIPAC-directed treatment

3224 PIPAC-directed treatments were performed and 33 % of the patients received PIPAC 1 as part of a prospective study.

The rate of nonaccess was 3.5 % in PIPAC 1 dropping to 1.4 % in subsequent PIPAC-directed treatments (p<0.01).

Bidirectional treatment, when defined as systemic chemotherapy within 4 weeks before PIPAC-directed treatments, was administered in 57 % of the patients. The number of patients who received bidirectional treatment did not change significantly between the first three PIPAC-directed treatments and subsequent treatments from PIPAC 4 (p=0.09).

Data from all PIPAC directed treatments and intraoperative findings are listed in Table 2 (Table 3, available as Supplementary data).

**Table 2:** Data from 3224 PIPAC-directed treatments.

PIPAC data	Total
Total number of PIPAC-directed treatments (%)	3224 (100)
Number of PIPAC per patient, median (range)	2 (1–33)
Nonaccess, n (%)	68 (2)
Electrostatic precipitation, n(%)	223 (7)
PCI^a^ score complete^b^, n (%)	2205 (83)
PCI score, median (range)	
PIPAC 1	19 (0–39)
All PIPAC treatments	19 (0–39)
Flowrate, n (%), n=681	
0.4–0.59 mL/s	319 (47)
0.6–0.79 mL/s	247 (36)
0.8–0.99 mL/s	99 (15)
≥1.0 mL/s	16 (2)
Exposure time, n (%), n=1084	
1 min	28 (3)
6 min	22 (2)
12 min	36 (3)
30 min	983 (91)
Histological response evaluation performed	
None	483 (15)
PRGS^c^	2306 (72)
Non-PRGS	322 (10)
Unknown/missing	113 (4)
Ascites, reported cases (%)	1394 (43)
Median volume, mL (range)	200 (1–19000)
Systemic chemotherapy within 4 weeks, n (%)	1501 (57)
Other surgical procedures, n (%)	76 (2)
Median length of stay (95 % percentile)	2 days (4 days)

PCI^a^, peritoneal cancer index; ^b^PCI was complete when all 13 different regions were visualized, PRGS^c^, peritoneal regression grading score.

Other surgical procedures (n=76) included partial peritonectomy (n=17), bowel resection (n=15), adnexectomy (n=9), omentectomy (n=3), and was not specified in 32 procedures.

Ascites was observed in 43 % of the procedures. The number of patients with an ascites volume between 500 and 1000 mL was significantly reduced between the first three PIPAC treatments and subsequent treatments from PIPAC 4 (p<0.01). The same was observed for patients with an ascites volume >1000 mL (p<0.01).

### Complications

Surgical complications and adverse events were reported in 1437 PIPAC-directed treatments (45 %), and details are listed in Table 3. Major surgical complications (DC ≥3b) occurred in 27 (0.8 %) of the treatments, while other adverse events with CTCAE grade ≥3 were reported in 168 treatments (5.2 %). Grade 1–3a surgical complications included bleeding (n=17), wound dehiscence (n=5), bowel injury (n=4), intra-abdominal abscess (n=3), ascites fistula (n=1), and other but not specified (n=58). Most frequent grade 3b complications were perforation (n=5), bowel injury (n=4), bleeding (n=2), and other but not specified (n=12). The two grade 4 and 5 surgical complications were not further described. No significant difference was observed in the rate of surgical complications or adverse events among patients with gastric, colon, ovarian, pancreas, or appendix cancers (p=0.77). Most frequent adverse events are listed in Table 3.

**Table 3:** All registered complications and adverse events in the ISSPP PIPAC database.

Complications and adverse events	Total
Total no. of PIPAC treatments (%)	3224 (100)
Number of detailed and graded events^a^	Surgical: 115 (3.6 %)
	Adverse events: 2735 (85 %)
Surgical complications^b^, n (%)	
Grade 1–3a	88 (77)
Grade 3b	25 (22)
Grade 4 and 5	2 (2)
Adverse events (CTCAE)^c^, n (%)	
Grade 1	1200 (44)
Grade 2	1367 (50)
Grade 3	156 (6)
Grade 4	4 (0.1)
Grade 5	8 (0.2)
Most common adverse events, n (%)	
Abdominal pain	1156 (42)
Nausea	358 (13)
Vomiting	261 (10)
Constipation	220 (8)
Urinary retention	129 (5)
Peripheral sensory neuropathy	90 (3)
Diarrhea	62 (2)

^a^A PIPAC treatment may be accompanied by multiple complications or adverse events. ^b^Dindo–Clavien classification. ^c^Common terminology criteria for adverse events version 5.0.

### Postoperative mortality

No postoperative mortality was observed within 1 day after PIPAC 1. The 30-day and 90-day mortality after PIPAC 1 was 1.15 % and 10.3 %, respectively. The 90-day mortality after PIPAC 1 was significantly higher in patients with gastric cancers (11.2 %), ovarian cancers (12.5 %), and pancreatic cancers (13.7 %) than in patients with colon cancers (4.0 %) and appendix cancers (3.0 %) (p<0.01).

Concerning the postoperative mortality after the last PIPAC, the mortality was 1/960 (0.1 %) within day 1, while the 30- and 90-day mortality from the last treatment was 3.23 % and 23.5 %, respectively. The 90-day mortality after the last treatment was significantly higher in patients with gastric cancer (31%) compared to patients with other primary tumors (p<0.01).

### Response evaluation

Peritoneal regression grading score (PRGS) was performed to 2306 (72 %) PIPAC-directed treatments. Overall mean PRGS was 2.21 at PIPAC 1 (n=784) and dropping to 1.87 at PIPAC 3 (n=376) and 1.68 at PIPAC 6 (n=80). A PRGS ≤2 or a reduction of the mean PRGS of at least 1 between PIPAC 1 and 3 was observed in 75 % of patients (n=404).

In total 57 %, 72 %, and 75 % had a mean PRGS ≤2 at PIPAC 1, 2, and 3, respectively. The number of patients with a complete/major response (mean PRGS ≤ 2) increased from PIPAC 1 to PIPAC 3 across the five most common primary tumor types (Table 4).

**Table 4:** The rate of patients with complete/major response (mean PRGS≤2) at PIPAC 1–3 stratified by primary tumor.

Primary tumor	PIPAC 1, n (%)	PIPAC 2, n (%)	PIPAC 3, n (%)
Gastric	177 (72)	160 (80)	121 (77)
Colon	80 (63)	71 (83)	60 (92)
Ovaries	40 (41)	37 (57)	35 (71)
Pancreas	29 (58)	24 (67)	16 (70)
Appendix	16 (47)	28 (76)	20 (74)

Non-peritoneal regression grading score (non-PRGS) was performed in 322 (10 %) PIPAC-directed treatments. A response evaluation was available in 189 of the 322 (59 %) treatments. Among the 189 response evaluations, no additional data was listed in 137 (72 %). No response was noted in 35 (19 %) treatments, partial response in 10 (5 %), and complete response in 7 (4 %). No response evaluation was performed in 483 PIPAC treatments (15 %), while data were missing in 113 (4 %) of the treatments (Table 2).

### Follow-up

The median follow-up time was 8.1 months (range 0–65 months). Reasons for stopping PIPAC were available 1006 of the 1126 patients (89 %). Disease progression was the major cause and listed in 515 (46 %) of the patients followed by poor general condition in 68 patients (6 %), patient refusal in 67 patients (6 %), and technical reasons in 64 patients (6 %). Curative intended surgery was the reason in 58 patients (5 %). Death and end of study were the reasons in 47 (4 %) and 28 (2), respectively. Other reasons accounted for 117 patients (14 %), and data were missing in 120 patients (11 %).

The location of disease progression was reported in 148 patients. Isolated peritoneal progression was the most frequent location (45 %) followed by PM plus extraperitoneal metastases (23 %) and isolated extraperitoneal metastases (20 %). Other locations were noted in 12 %.

### Survival

A total of 725 patients (64 %) were registered as dead. The validity of follow-up when measuring true registration of death was 59 % (range 0–100).

Median overall survival from PIPAC 1 was 12.5 months (n=1060, 95 % CI; [11.3–13.6]).

The median overall survival stratified by primary tumor with at least 10 patients is shown in Table 5. Among these, the proportion of patients receiving ≥3 PIPAC-directed treatments was 46.4 %.

**Table 5:** The number of patients receiving ≥3 PIPACs and median overall survival from PIPAC 1 according to primary tumor, where at least 10 patients were treated (n=1072).

Primary tumor	Total patients, n	No of patients receiving ≥3 PIPACs, n (%)	Median overall survival, months (95 % CI)
Gastric	404	207 (51)	9.9 (8.7–11.1)
Colon	181	80 (44)	16.0 (12.6–20.5)
Ovaries	141	64 (45)	14.5 (9.6–17.6)
Pancreas	91	29 (32)	10.9 (8.2–13.1)
Appendix	79	40 (51)	19.8 (15.8–47.6)
Mesothelioma	62	38 (61)	33.5 (14.1–)
Bile duct	47	19 (40)	12.0 (7.9–18.3)
Small bowel	19	11 (58)	12.5 (7.3–14.4)
Rectum	17	4 (24)	9.3 (4.5–)
Breast	16	7 (44)	19.2 (5.6–)
Primary peritoneal	15	9 (60)	9.4 (5.6–16.3)

### Survival and PRGS

**Figure 2:**
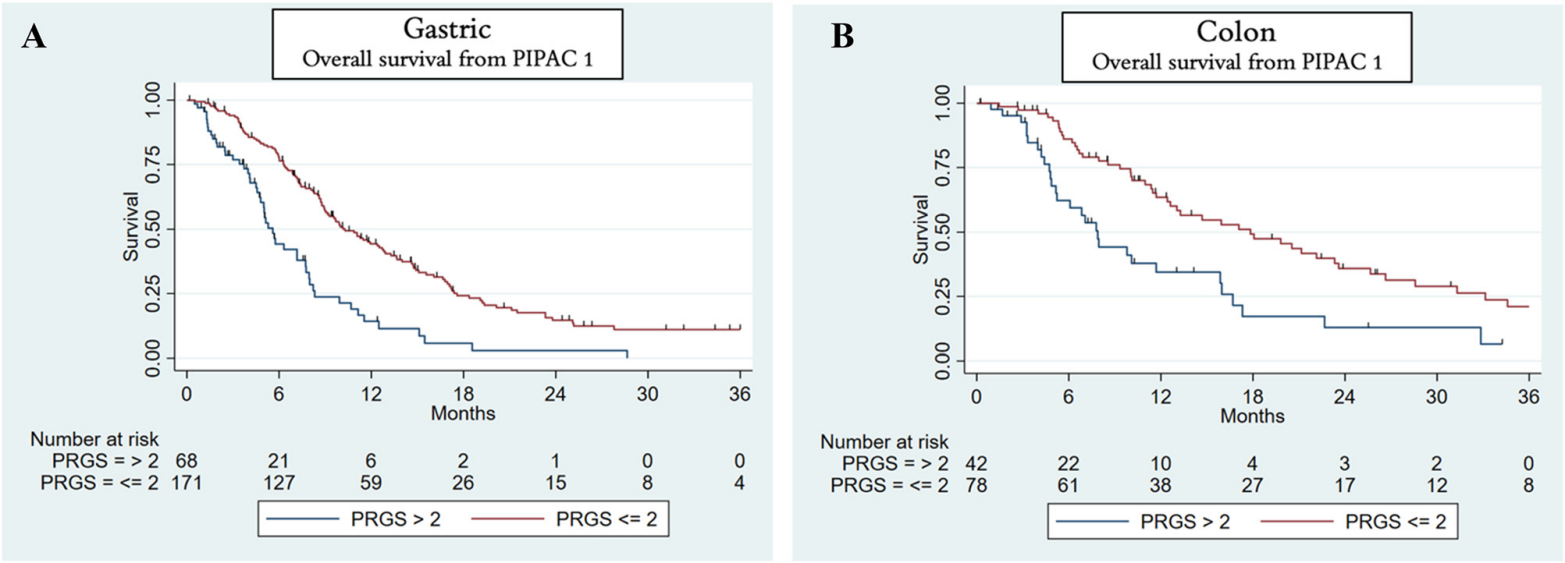
Overall survival based on peritoneal regression grading score (PRGS) at PIPAC 1. Red curve shows patients with complete/major response (mean PRGS ≤2), and blue curve shows patients with minimal/no response (mean PRGS >2) for patients with gastric cancer (A) and colon cancer (B).

**Figure 3:**
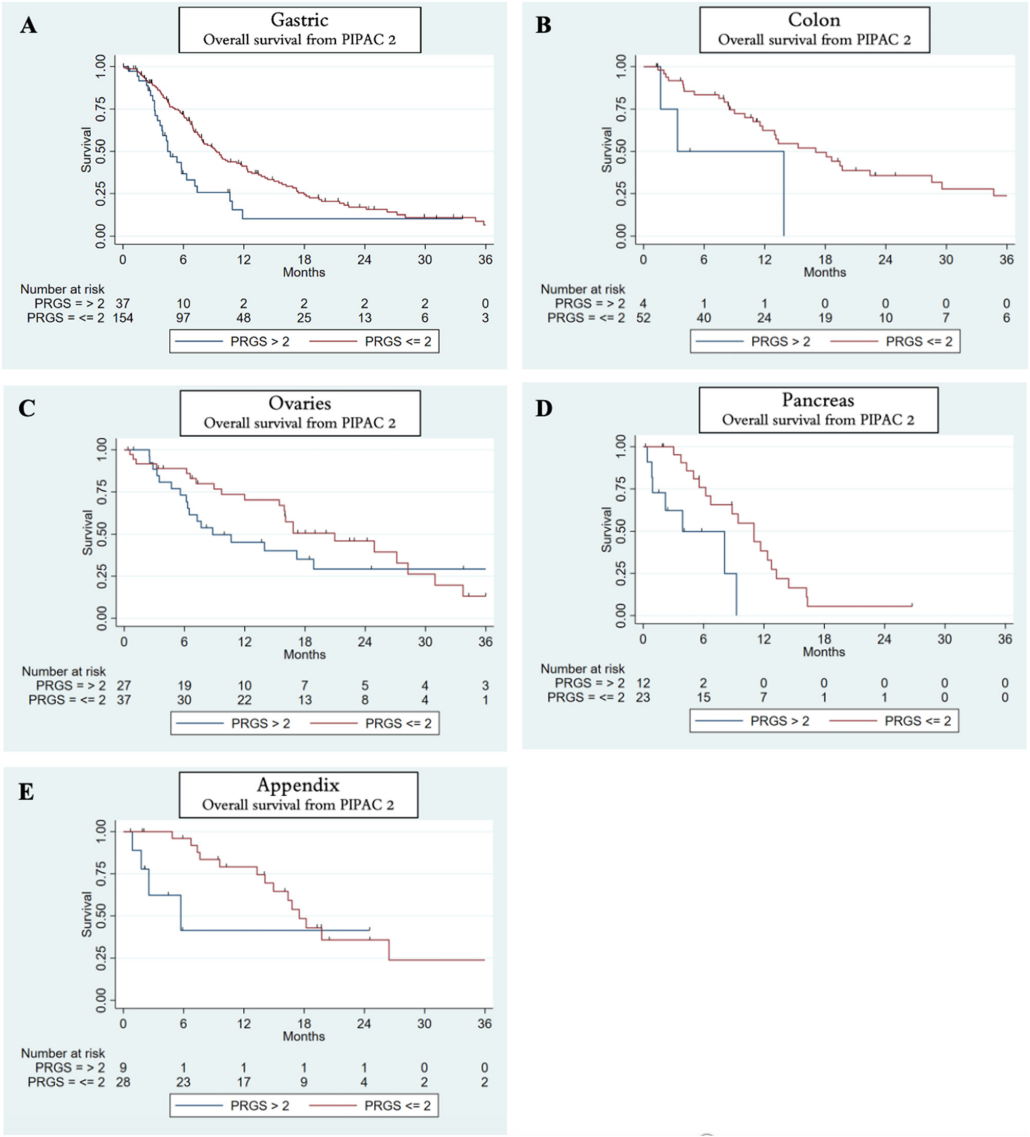
Overall survival based on peritoneal regression grading score (PRGS) at PIPAC 2. Red curve shows patients with complete/major response (mean PRGS ≤2), and blue curve shows patients with minimal/no response (mean PRGS >2) for patients with gastric cancer, colon cancer, ovary cancer, pancreas cancer, or appendix cancer (A–E).

Patients with gastric cancer or colon cancer with mean PRGS ≤2 at PIPAC 1 had a longer overall survival than patients with mean PRGS >2 (Figure 2). A similar observation was made at PIPAC 2 (Figure 3) and PIPAC 3 (Figure 4, available as Supplementary data) for patients with gastric cancer, colon cancer, ovarian cancer, pancreatic cancer, and appendix cancers. No Kaplan–Meier analysis was performed in patients with colon cancers at PIPAC 3 due to no patients with minimal/no response (mean PRGS >2) (Figure 4, available as Supplementary data).

## Discussion

The ISSPP PIPAC database is the world’s largest, noncommercial, voluntary, quality assuring PIPAC database with data from 1126 patients and 3224 PIPAC-directed treatments as of November 1st, 2024. Patients with PM from different primary tumors have been included since the official launch of the database in June 2020, and data from the first 809 patients were reported in the second annual report in 2023 [5]. Since 2023, the number of reporting PIPAC centers has increased by five, and today 31 PIPAC centers are registered as contributors and 17 are actively submitting patient data to the database.

As expected, and consistent with the findings of the second annual report, the results from this study align with recent systematic reviews with regard to the characteristics of the study population, the distribution of primary tumors, the number of PIPAC-directed treatments received by patients, and the PCI scores [10], 11]. Furthermore, the rates of nonaccess, chemotherapy dosage, flow rates, and exposure times are also consistent with existing literature [11], 12]. Variations observed regarding the chemotherapy dosage, flow rates, and exposure times may be attributed to the evolution of PIPAC-directed treatments (e.g., recommended doses) over time, variations between reporting centers as well as the introduction of new aerosolizers.

The complications and adverse events data recorded in the ISSPP PIPAC database also mirror the general opinion that patients scheduled for PIPAC are well selected and thus ensuring a satisfactory safety and feasibility profile [11], 12]. Also, the postoperative mortality within day 1 (0.1 %) and the 30-day mortality from last treatment (3 %) seem to align with findings in the second annual report and in two recent systematic reviews [5], 11], 13].

Patients with PM from gastric cancer had a 90-day mortality after last PIPAC-directed treatment of 31 %, which was significantly higher than for patients with other primary tumors. However, with 11.2 %, their 90-day mortality following PIPAC 1 was comparable to the mortality observed in patients with ovarian or pancreatic cancers [10], 11]. Ninety days mortality is rarely reported in PIPAC studies, but one study recorded 17 % among patients with gastric cancer treated with a bidirectional approach, and cause of death was reported to have been mainly due to disease progression and to not have been related to the PIPAC-directed treatment [14]. Data on cause of death are not available in the ISSPP PIPAC database, but since there was no difference in the rate of complications or adverse events between patients with different primary tumors, the high 90-day mortality after the last treatment reported here is probably unrelated to the PIPAC-directed treatments and instead may reflect the poor prognosis of gastric cancer patients with PM [12], 14]. Careful selection of all PM patients for PIPAC-directed therapy is still a topic of great importance. The minimum expected survival time at the time of inclusion (i.e., PIPAC 1) may vary between PIPAC centers and trials, but three out of four patients surviving at least 3 months after the last PIPAC-directed treatment seems to justify the present patient selection. One must, however, consider that pooling data from patients with different primary tumors might introduce biases and challenges for the interpretation of the results.

PRGS seems able to reflect the state of PM at different time points and thus the potential local treatment effect during PIPAC-directed or bidirectional therapy [15]. PRGS was the most commonly used tool for response evaluation, and throughout PIPAC 1–3 a mean PRGS ≤2 (i.e., a complete or major response) was observed in 57 %, 72 %, and 75 % of patients, respectively. This is comparable to data from a recent review investigating response evaluation in patients with PM treated with PIPAC, which reported a stable or improved PRGS in 18–58 % of patients [16]. The large variation in the review was due to heterogeneity of patients or a high number of discontinued treatments in the included studies [16]. The higher response rates observed in the present study may be explained by a larger study population, a more selected patient cohort, or other contributing factors.

The data we show demonstrates, that patients with PM from gastric, colon, ovarian, pancreatic, or appendix cancers, who have a mean PRGS ≤2 at PIPAC 1, 2, or 3 seem to have a longer overall survival compared with patients who have a PRGS>2. Similar observations were made in a large prospective phase II trial [15], while other studies found PRGS not to be an independent prognostic factor [17], 18]. This demonstrates that patients responding to systemic chemotherapy and PIPAC have a better prognosis. The isolated effect of PIPAC-directed treatments remains unclear, and randomized controlled trials are needed to investigate this topic. Available data suggest that PRGS and index PCI might be markers of local treatment response, but the definitive prognostic value and role of PRGS, however, requires further investigation [16].

The most frequent reason for stopping PIPAC was disease progression followed by poor general condition, and patient refusal. This was unchanged compared to the second annual PIPAC report and consistent with a recent systematic review, which reported disease progression to be the reason for stopping PIPAC in nearly 50 % of patients [10]. To provide more specific data on progression, a new database variable (location of disease progression) was launched in July 2024, and data from the first 148 patients found peritoneum to be the most frequent location of disease progression. To the best of our knowledge, this topic has not been previously explored, underscoring the need for further research.

This study shows encouraging survival results following PIPAC-directed treatment, and the results are in line with recent systematic reviews [12], 13], 19]. In addition, PIPAC and systemic chemotherapy may result in patients being long-term survivors [20], or even to be able to undergo radical resection [10], 21]. Patients with peritoneal mesothelioma, in particular, appear to benefit from PIPAC, with a median overall survival (OS) of 33.5 months [22]. This represents the longest OS ever reported for peritoneal mesothelioma patients receiving PIPAC and is nearly comparable to outcomes achieved with a combination of cytoreductive surgery and Hyperthermic Intraoperative Peritoneal Chemotherapy (HIPEC) [23], 24]. However, the results from systematic reviews and the ISSPP PIPAC database should be interpreted with caution due to heterogeneity regarding inclusion criteria and treatment strategies [12], 13], 19].

Recent studies suggest an increased survival with the numbers of delivered PIPAC treatments [11], 12]. In the present study, almost half of the patients had three or more PIPAC-directed treatments irrespective of their primary cancer type, and this may reflect good patient selection, treatment response, or probably a mixture of several known and unknown factors.

### Limitations

This study has several limitations, and its primary limitation is missing or incomplete data, which was also seen in the second annual report. However, most major databases must and can accept missing data as long as a steady improvement occurs over time. Some missing data arise from incorrect registration, which may be corrected by optimizing the database and improving concomitant guidelines.

Another limitation is the decline in follow-up validity to 59 % compared to 71 % in the second annual report. One possible explanation for this decline is that one major reporting PIPAC center has closed and is no longer performing follow-up. Additionally, other centers that have enrolled a large number of patients may lack the resources or the capacity necessary to update patient follow-up data on an annual basis.

In terms of external validity, the four largest PIPAC centers accounted for 77 % of the patient cohort, representing an improvement from the 93 % observed in 2023. While this improvement suggests a broader inclusion of patients, the results of this study continue to (mainly) reflect the combined outcomes of these four major PIPAC centers.

Additionally, this study is descriptive and based on data from an exploratory database primarily established for monitoring purposes. Future scientific studies based on data from the ISSPP PIPAC should include specific and predefined outcomes with relevant statistical testing to explore significant associations.

### New initiatives

During the ISSPP/PSOGI 14th International congress on Peritoneal Surface Malignancies in Lyon, France, in September 2024, the ISSPP Registry Group decided on a series of initiatives to improve the ISSPP PIPAC database. First, as a step to reduce workload during data registration, it was decided that only major adverse events (CTCAE≥3) will be registered in the future, whereas all surgical complications will still be noted. Second, to promote follow and improve data completeness in general, it was also agreed, that a minimum of five new patients per year should be registered with a complete data set in order to fulfill criteria to be an active center within the ISSPP PIPAC database; in addition, a detailed analysis of data completeness within the database shall be reported separately. Last, several new initiatives have been devised to maintain and improve the ISSPP PIPAC database: Odense PIPAC Center and ISSPP launched a support program with a help desk (ouh.a.pipac@rsyd.dk) that sends requests for data corrections to reporting centers, updates recommendations for data inclusion, and creates and maintains more helpful in-database instructions. The ISSPP PIPAC Registry group hopes that the increased focus and additional practical and technical initiatives and improvements may further improve the data and thus the value of the ISSPP PIPAC database.

## Conclusion

The third annual report from the ISSPP PIPAC database provides important real-world data supporting the use, safety, and effect of PIPAC-directed therapy in patients with PM from different primary tumors. Data from the ISSPP PIPAC database align with existing literature and suggest that PIPAC combined with systemic chemotherapy can induce local tumor responses and potentially prolong survival in selected cancer patients. Continuous monitoring and registration of PIPAC data in the ISSPP PIPAC database remains essential while awaiting data from randomized controlled trials

## Supplementary Material

Supplementary Material
