# Supplementary material for: Real-world data on Pressurized IntraPeritoneal Aerosol Chemotherapy (PIPAC)-directed therapy in patients with peritoneal metastases; Third annual report from the ISSPP PIPAC database
Source: Pleura Peritoneum. 2025 Jun 10;10(3):119–28. doi: 10.1515/pp-2025-0013 (PMC12458025; doi:10.1515/pp-2025-0013)
Supplement: Supplementary file 1 — Supplementary Material [file j_pp-2025-0013_suppl_001.docx]

**Supplementary material**

**Supplementary A:**

**Table 3** Reported drugs and doses per unit of body surface area of oxaliplatin, cisplatin and doxorubicin during PIPAC 1.

| **Drugs (n)** | | **Doses*** | | **n (%)** |
| --- | --- | --- | --- | --- |
| Oxaliplatin (n=256) | | <90 mg/m^2^ | | 5 (2) |
|  | | 90 mg/m^2^ | | 12 (5) |
|  | | 92 mg/m^2^ | | 200 (78) |
|  | | ≥92.5 mg/m^2^ | | 39 (15) |
| Cisplatin (n=794) | | <7.5 mg/m^2^ | | 2 (1) |
|  | | 7.5 mg/m^2^ | | 349 (43) |
|  | | 10.5 mg/m^2^ | | 420 (53) |
|  | | >10.5 mg/m^2^ | | 23 (3) |
| Doxorubicin (n=757) | | 1.5 mg/m^2^ | | 345 (46) |
|  | | 2.1 mg/m^2^ | | 385 (51) |
|  | | Other | | 27 (4) |
| Mitomycin C (n=10) | |  | |  |
| Nab-paclitaxel (n=7) | |  | |  |
| Other (n=9) | |  | |  |
| * Per unit of body surface area | | |  |  |
|  |  |  |  |  |

**Supplementary B**:

**
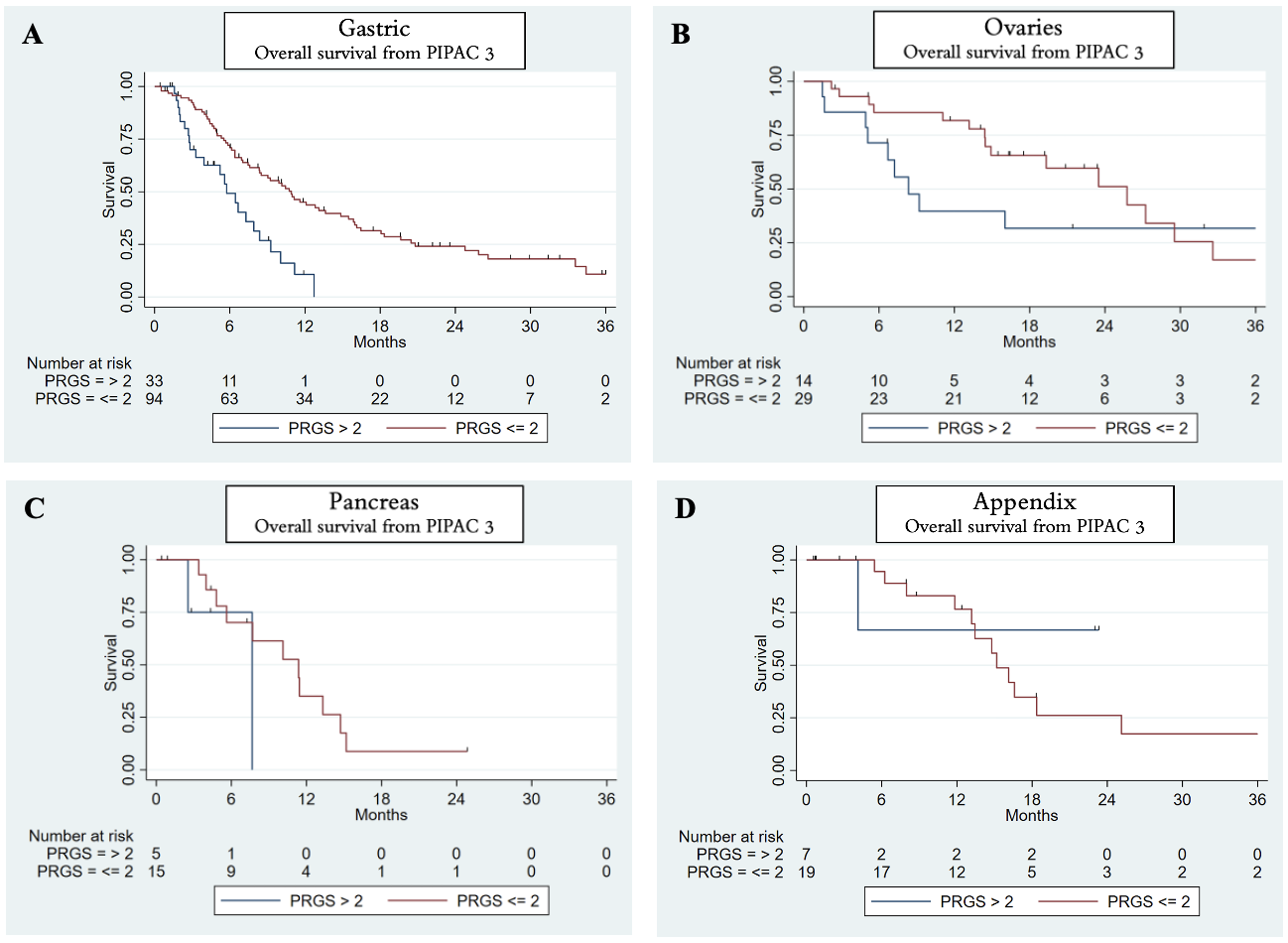
**

**Figure 4** Overall survival based on peritoneal regression grading score (PRGS) at PIPAC 2. Red curve shows patients with complete/major response (mean PRGS ≤2) and blue curve shows patients with minimal/no response (mean PRGS >2) for patients with gastric cancer, ovary cancer, pancreas cancer or appendix cancer (A-C).
